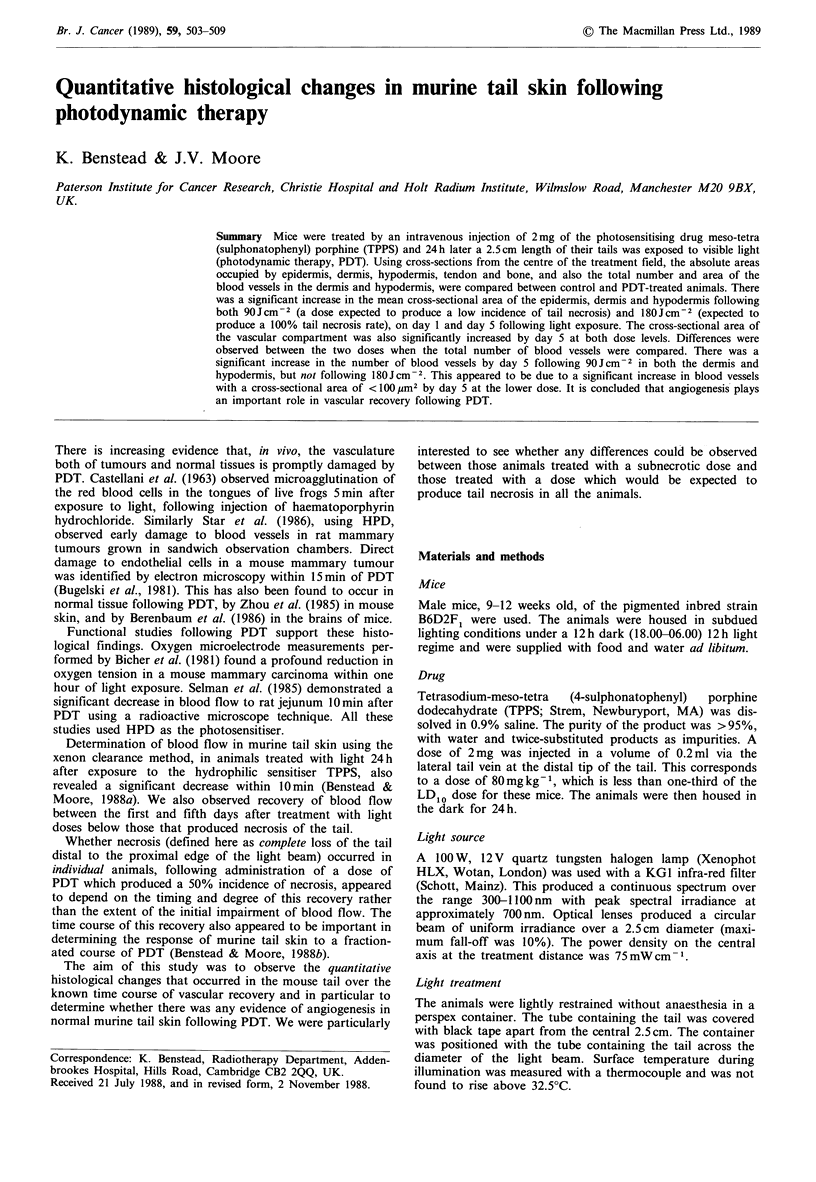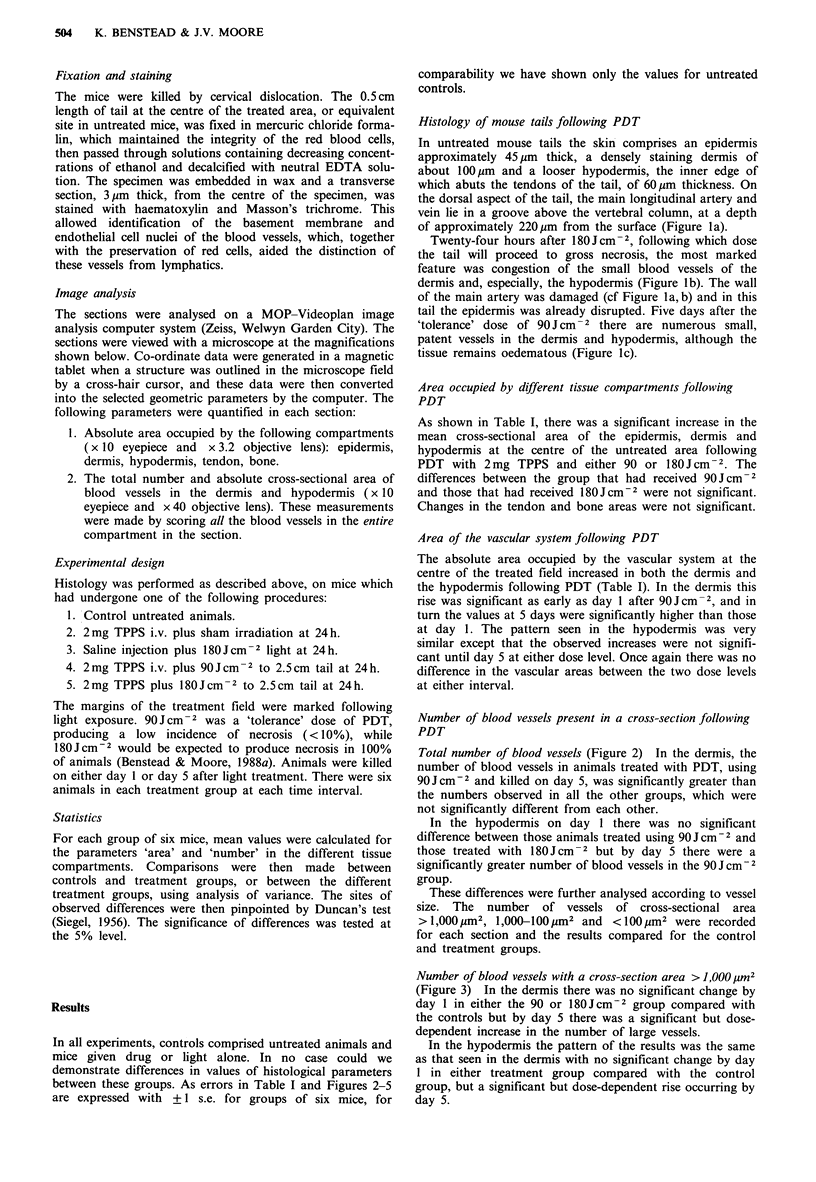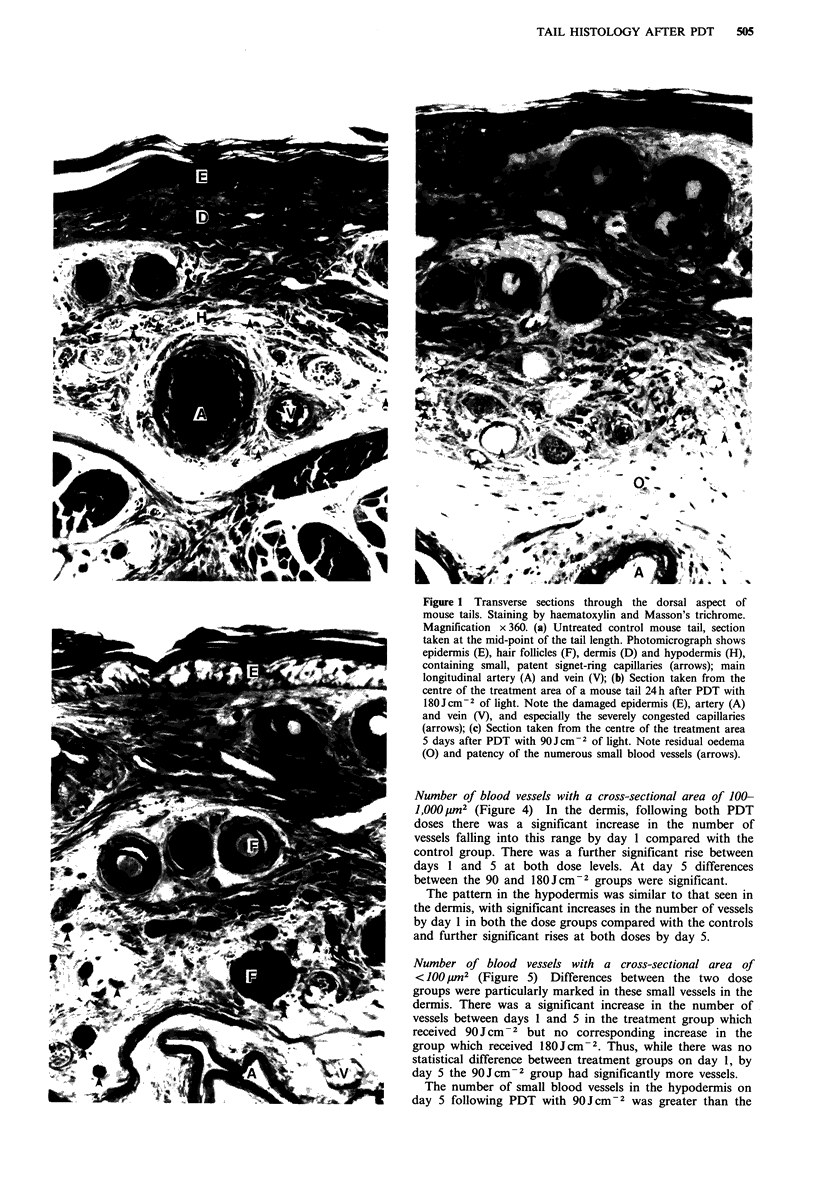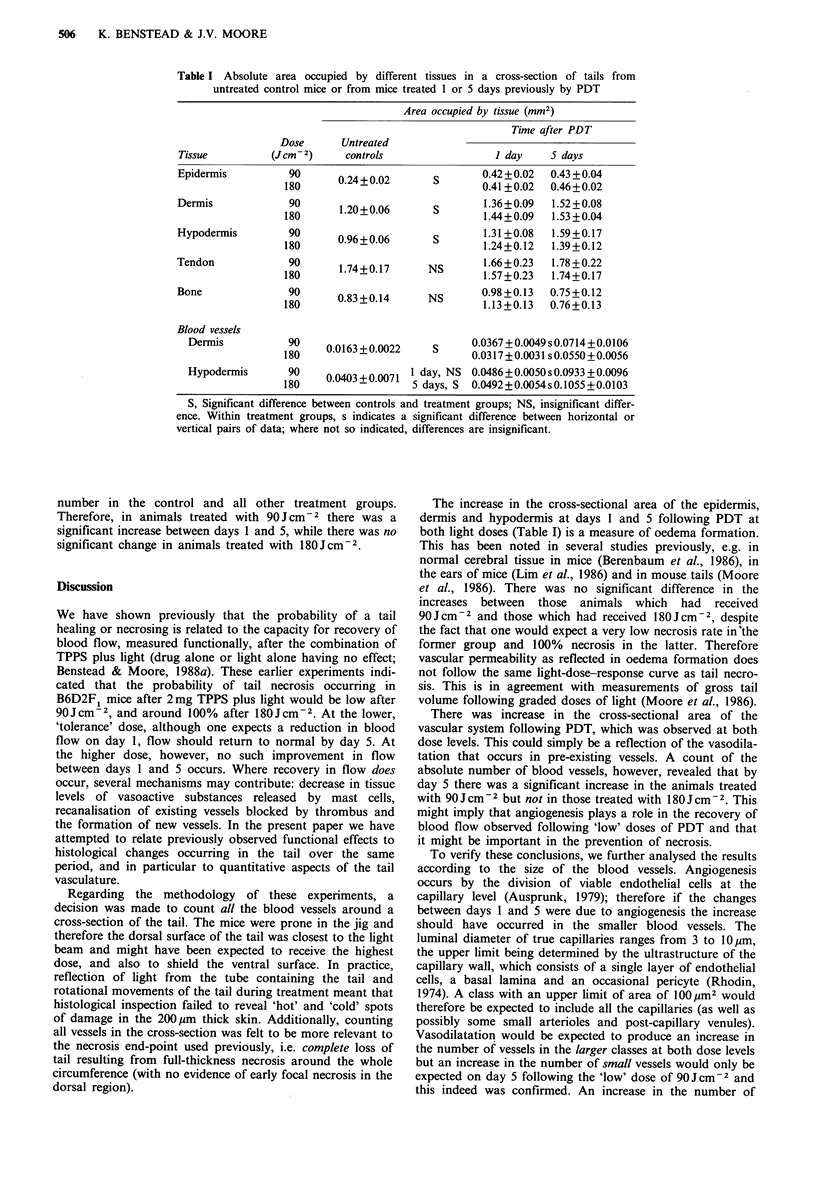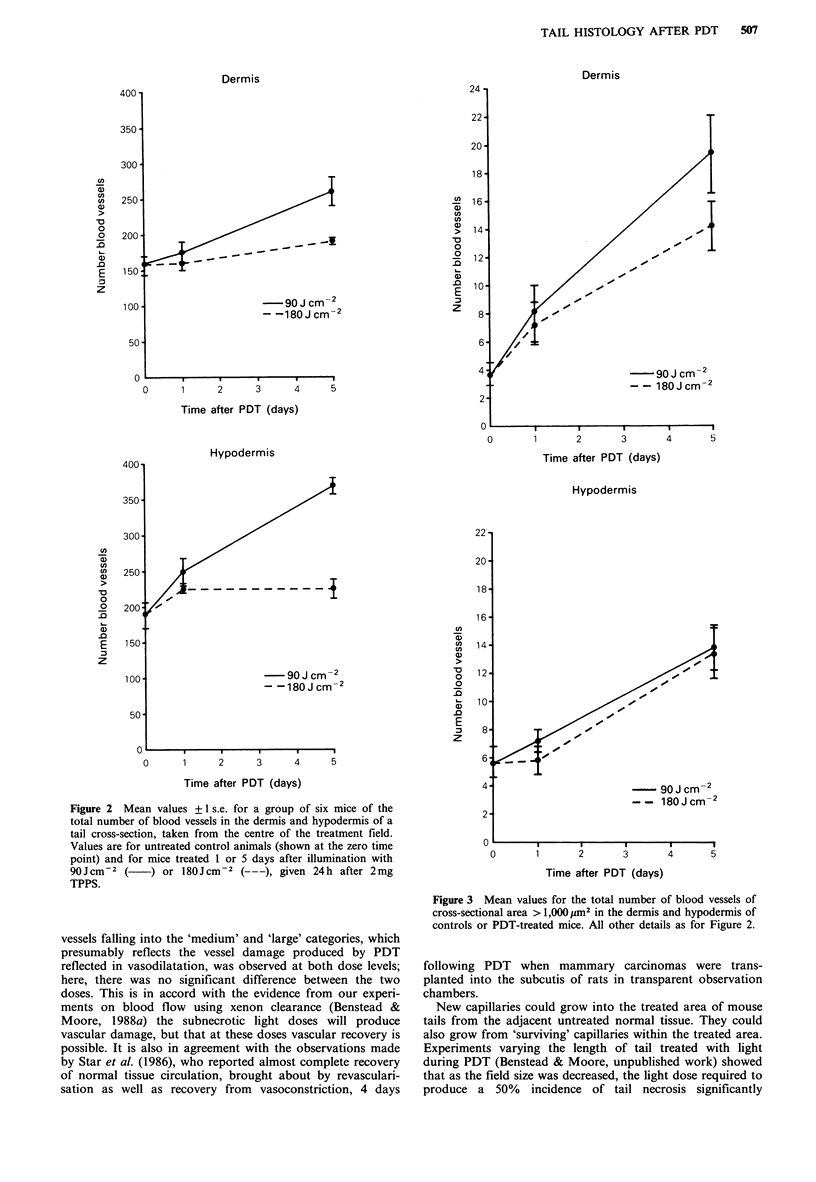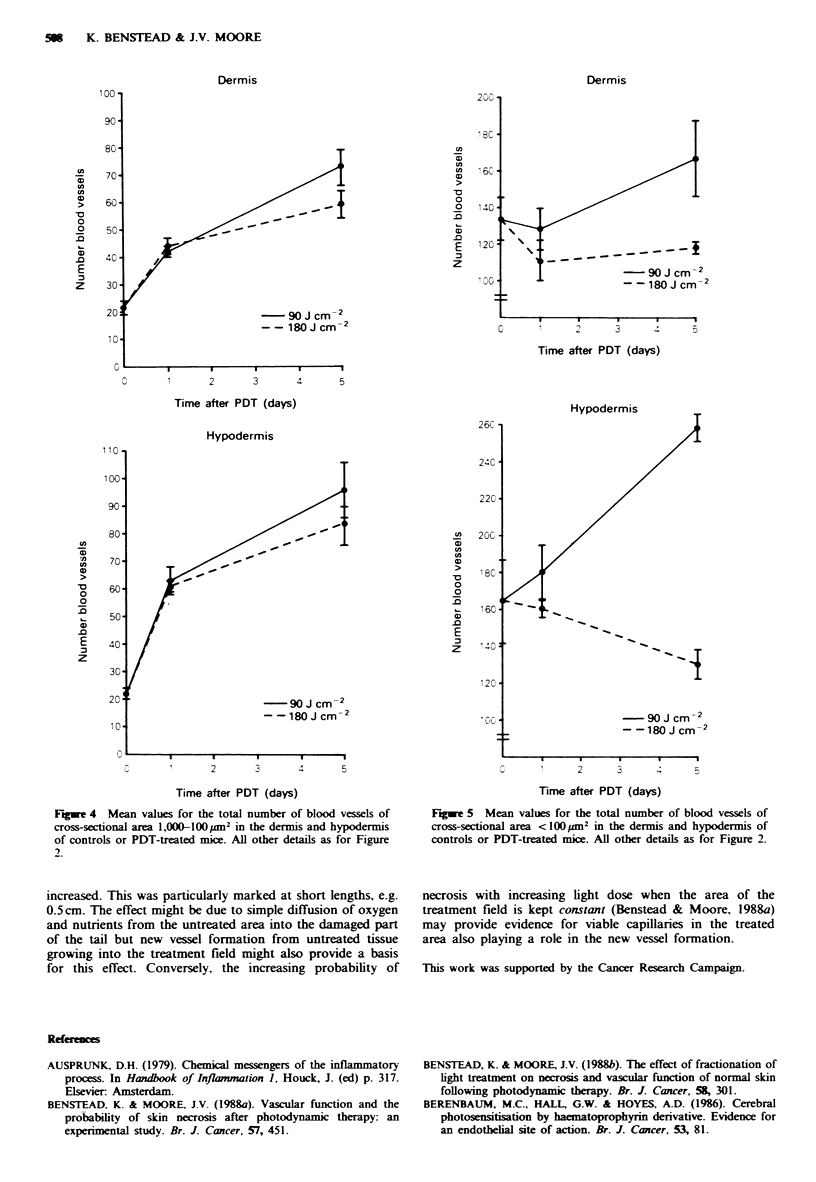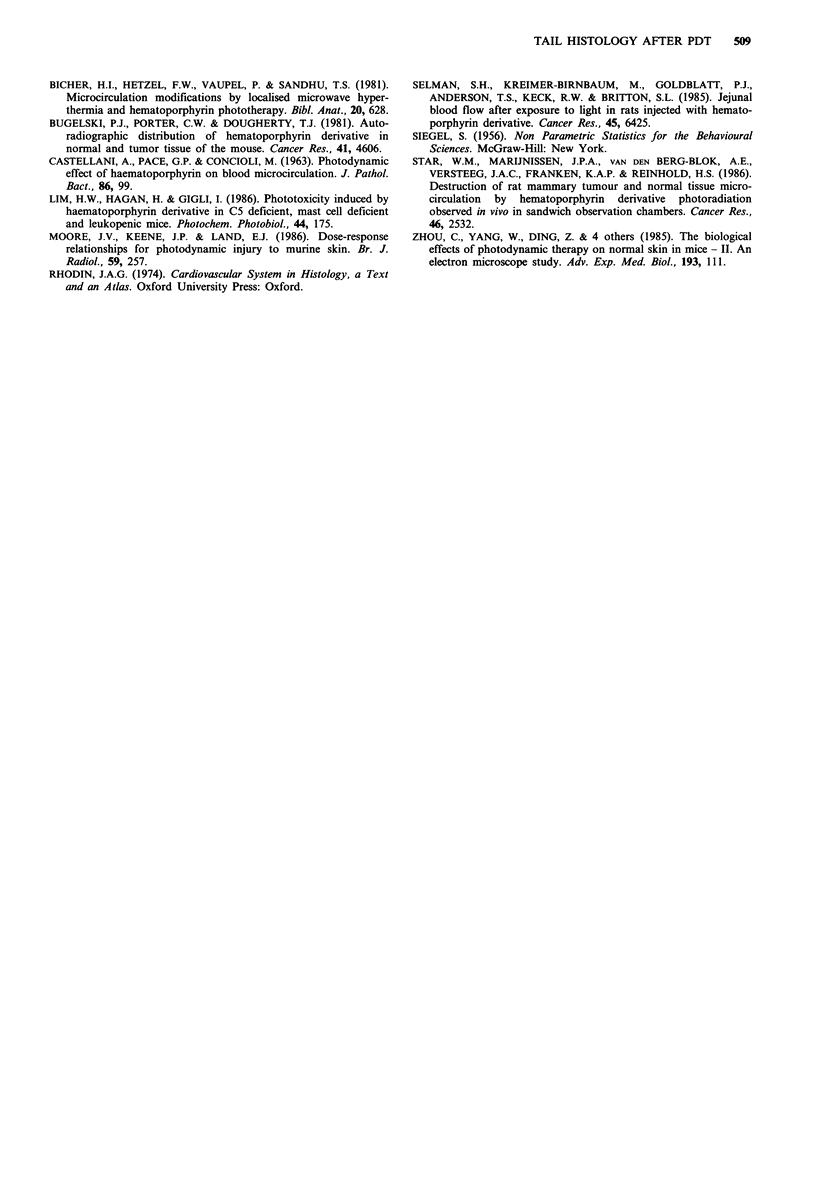# Quantitative histological changes in murine tail skin following photodynamic therapy.

**DOI:** 10.1038/bjc.1989.104

**Published:** 1989-04

**Authors:** K. Benstead, J. V. Moore

**Affiliations:** Paterson Institute for Cancer Research, Christie Hospital and Holt Radium Institute, Manchester.

## Abstract

**Images:**


*Br. J. Cancer* (1989), **59**, 503–509 © The Macmillan Press Ltd., 1989

### Quantitative histological changes in murine tail skin following photodynamic therapy

K. Benstead & J.V. Moore

*Paterson Institute for Cancer Research, Christie Hospital and Holt Radium Institute, Wilmslow Road, Manchester M20 9BX, UK.*

**Summary** Mice were treated by an intravenous injection of 2 mg of the photosensitising drug meso-tetra (sulphonatophenyl) porphine (TPPS) and 24 h later a 2.5 cm length of their tails was exposed to visible light (photodynamic therapy, PDT). Using cross-sections from the centre of the treatment field, the absolute areas occupied by epidermis, dermis, hypodermis, tendon and bone, and also the total number and area of the blood vessels in the dermis and hypodermis, were compared between control and PDT-treated animals. There was a significant increase in the mean cross-sectional area of the epidermis, dermis and hypodermis following both 90 J $cm^{-2}$ (a dose expected to produce a low incidence of tail necrosis) and 180 J $cm^{-2}$ (expected to produce a 100% tail necrosis rate), on day 1 and day 5 following light exposure. The cross-sectional area of the vascular compartment was also significantly increased by day 5 at both dose levels. Differences were observed between the two doses when the total number of blood vessels were compared. There was a significant increase in the number of blood vessels by day 5 following 90 J $cm^{-2}$ in both the dermis and hypodermis, but *not* following 180 J $cm^{-2}$. This appeared to be due to a significant increase in blood vessels with a cross-sectional area of $<100\,\mu m^2$ by day 5 at the lower dose. It is concluded that angiogenesis plays an important role in vascular recovery following PDT.

There is increasing evidence that, *in vivo*, the vasculature both of tumours and normal tissues is promptly damaged by PDT. Castellani *et al.* (1963) observed microagglutination of the red blood cells in the tongues of live frogs 5 min after exposure to light, following injection of haematoporphyrin hydrochloride. Similarly Star *et al.* (1986), using HPD, observed early damage to blood vessels in rat mammary tumours grown in sandwich observation chambers. Direct damage to endothelial cells in a mouse mammary tumour was identified by electron microscopy within 15 min of PDT (Bugelski *et al.*, 1981). This has also been found to occur in normal tissue following PDT, by Zhou *et al.* (1985) in mouse skin, and by Berenbaum *et al.* (1986) in the brains of mice.

Functional studies following PDT support these histological findings. Oxygen microelectrode measurements performed by Bicher *et al.* (1981) found a profound reduction in oxygen tension in a mouse mammary carcinoma within one hour of light exposure. Selman *et al.* (1985) demonstrated a significant decrease in blood flow to rat jejunum 10 min after PDT using a radioactive microscope technique. All these studies used HPD as the photosensitiser.

Determination of blood flow in murine tail skin using the xenon clearance method, in animals treated with light 24 h after exposure to the hydrophilic sensitiser TPPS, also revealed a significant decrease within 10 min (Benstead & Moore, 1988*a*). We also observed recovery of blood flow between the first and fifth days after treatment with light doses below those that produced necrosis of the tail.

Whether necrosis (defined here as *complete* loss of the tail distal to the proximal edge of the light beam) occurred in *individual* animals, following administration of a dose of PDT which produced a 50% incidence of necrosis, appeared to depend on the timing and degree of this recovery rather than the extent of the initial impairment of blood flow. The time course of this recovery also appeared to be important in determining the response of murine tail skin to a fractionated course of PDT (Benstead & Moore, 1988*b*).

The aim of this study was to observe the *quantitative* histological changes that occurred in the mouse tail over the known time course of vascular recovery and in particular to determine whether there was any evidence of angiogenesis in normal murine tail skin following PDT. We were particularly interested to see whether any differences could be observed between those animals treated with a subnecrotic dose and those treated with a dose which would be expected to produce tail necrosis in all the animals.

Correspondence: K. Benstead, Radiotherapy Department, Addenbrookes Hospital, Hills Road, Cambridge CB2 2QQ, UK.
Received 21 July 1988, and in revised form, 2 November 1988.

### Materials and methods

### *Mice*

Male mice, 9–12 weeks old, of the pigmented inbred strain $B6D2F_1$ were used. The animals were housed in subdued lighting conditions under a 12 h dark (18.00–06.00) 12 h light regime and were supplied with food and water *ad libitum*.

### *Drug*

Tetrasodium-meso-tetra (4-sulphonatophenyl) porphine dodecahydrate (TPPS; Strem, Newburyport, MA) was dissolved in 0.9% saline. The purity of the product was >95%, with water and twice-substituted products as impurities. A dose of 2 mg was injected in a volume of 0.2 ml via the lateral tail vein at the distal tip of the tail. This corresponds to a dose of 80 mg $kg^{-1}$, which is less than one-third of the $LD_{10}$ dose for these mice. The animals were then housed in the dark for 24 h.

### *Light source*

A 100 W, 12 V quartz tungsten halogen lamp (Xenophot HLX, Wotan, London) was used with a KG1 infra-red filter (Schott, Mainz). This produced a continuous spectrum over the range 300–1100 nm with peak spectral irradiance at approximately 700 nm. Optical lenses produced a circular beam of uniform irradiance over a 2.5 cm diameter (maximum fall-off was 10%). The power density on the central axis at the treatment distance was 75 mW $cm^{-1}$.

### *Light treatment*

The animals were lightly restrained without anaesthesia in a perspex container. The tube containing the tail was covered with black tape apart from the central 2.5 cm. The container was positioned with the tube containing the tail across the diameter of the light beam. Surface temperature during illumination was measured with a thermocouple and was not found to rise above 32.5°C.

*Fixation and staining*

The mice were killed by cervical dislocation. The 0.5 cm length of tail at the centre of the treated area, or equivalent site in untreated mice, was fixed in mercuric chloride formalin, which maintained the integrity of the red blood cells, then passed through solutions containing decreasing concentrations of ethanol and decalcified with neutral EDTA solution. The specimen was embedded in wax and a transverse section, 3 $\mu m$ thick, from the centre of the specimen, was stained with haematoxylin and Masson's trichrome. This allowed identification of the basement membrane and endothelial cell nuclei of the blood vessels, which, together with the preservation of red cells, aided the distinction of these vessels from lymphatics.

*Image analysis*

The sections were analysed on a MOP–Videoplan image analysis computer system (Zeiss, Welwyn Garden City). The sections were viewed with a microscope at the magnifications shown below. Co-ordinate data were generated in a magnetic tablet when a structure was outlined in the microscope field by a cross-hair cursor, and these data were then converted into the selected geometric parameters by the computer. The following parameters were quantified in each section:

1. Absolute area occupied by the following compartments ($\times$10 eyepiece and $\times$3.2 objective lens): epidermis, dermis, hypodermis, tendon, bone.
2. The total number and absolute cross-sectional area of blood vessels in the dermis and hypodermis ($\times$10 eyepiece and $\times$40 objective lens). These measurements were made by scoring *all* the blood vessels in the *entire* compartment in the section.

*Experimental design*

Histology was performed as described above, on mice which had undergone one of the following procedures:

1. Control untreated animals.
2. 2 mg TPPS i.v. plus sham irradiation at 24 h.
3. Saline injection plus 180 J cm$^{-2}$ light at 24 h.
4. 2 mg TPPS i.v. plus 90 J cm$^{-2}$ to 2.5 cm tail at 24 h.
5. 2 mg TPPS plus 180 J cm$^{-2}$ to 2.5 cm tail at 24 h.

The margins of the treatment field were marked following light exposure. 90 J cm$^{-2}$ was a 'tolerance' dose of PDT, producing a low incidence of necrosis ($<$10%), while 180 J cm$^{-2}$ would be expected to produce necrosis in 100% of animals (Benstead & Moore, 1988*a*). Animals were killed on either day 1 or day 5 after light treatment. There were six animals in each treatment group at each time interval.

*Statistics*

For each group of six mice, mean values were calculated for the parameters 'area' and 'number' in the different tissue compartments. Comparisons were then made between controls and treatment groups, or between the different treatment groups, using analysis of variance. The sites of observed differences were then pinpointed by Duncan's test (Siegel, 1956). The significance of differences was tested at the 5% level.

### Results

In all experiments, controls comprised untreated animals and mice given drug or light alone. In no case could we demonstrate differences in values of histological parameters between these groups. As errors in Table I and Figures 2–5 are expressed with $\pm$1 s.e. for groups of six mice, for comparability we have shown only the values for untreated controls.

*Histology of mouse tails following PDT*

In untreated mouse tails the skin comprises an epidermis approximately 45 $\mu m$ thick, a densely staining dermis of about 100 $\mu m$ and a looser hypodermis, the inner edge of which abuts the tendons of the tail, of 60 $\mu m$ thickness. On the dorsal aspect of the tail, the main longitudinal artery and vein lie in a groove above the vertebral column, at a depth of approximately 220 $\mu m$ from the surface (Figure 1a).

Twenty-four hours after 180 J cm$^{-2}$, following which dose the tail will proceed to gross necrosis, the most marked feature was congestion of the small blood vessels of the dermis and, especially, the hypodermis (Figure 1b). The wall of the main artery was damaged (cf Figure 1a, b) and in this tail the epidermis was already disrupted. Five days after the 'tolerance' dose of 90 J cm$^{-2}$ there are numerous small, patent vessels in the dermis and hypodermis, although the tissue remains oedematous (Figure 1c).

*Area occupied by different tissue compartments following PDT*

As shown in Table I, there was a significant increase in the mean cross-sectional area of the epidermis, dermis and hypodermis at the centre of the untreated area following PDT with 2 mg TPPS and either 90 or 180 J cm$^{-2}$. The differences between the group that had received 90 J cm$^{-2}$ and those that had received 180 J cm$^{-2}$ were not significant. Changes in the tendon and bone areas were not significant.

*Area of the vascular system following PDT*

The absolute area occupied by the vascular system at the centre of the treated field increased in both the dermis and the hypodermis following PDT (Table I). In the dermis this rise was significant as early as day 1 after 90 J cm$^{-2}$, and in turn the values at 5 days were significantly higher than those at day 1. The pattern seen in the hypodermis was very similar except that the observed increases were not significant until day 5 at either dose level. Once again there was no difference in the vascular areas between the two dose levels at either interval.

*Number of blood vessels present in a cross-section following PDT*

*Total number of blood vessels* (Figure 2) In the dermis, the number of blood vessels in animals treated with PDT, using 90 J cm$^{-2}$ and killed on day 5, was significantly greater than the numbers observed in all the other groups, which were not significantly different from each other.

In the hypodermis on day 1 there was no significant difference between those animals treated using 90 J cm$^{-2}$ and those treated with 180 J cm$^{-2}$ but by day 5 there were a significantly greater number of blood vessels in the 90 J cm$^{-2}$ group.

These differences were further analysed according to vessel size. The number of vessels of cross-sectional area $>$1,000 $\mu m^2$, 1,000–100 $\mu m^2$ and $<$100 $\mu m^2$ were recorded for each section and the results compared for the control and treatment groups.

*Number of blood vessels with a cross-section area $>$1,000 $\mu m^2$* (Figure 3) In the dermis there was no significant change by day 1 in either the 90 or 180 J cm$^{-2}$ group compared with the controls but by day 5 there was a significant but dose-dependent increase in the number of large vessels.

In the hypodermis the pattern of the results was the same as that seen in the dermis with no significant change by day 1 in either treatment group compared with the control group, but a significant but dose-dependent rise occurring by day 5.

**Figure 1** Transverse sections through the dorsal aspect of mouse tails. Staining by haematoxylin and Masson's trichrome. Magnification ×360. (**a**) Untreated control mouse tail, section taken at the mid-point of the tail length. Photomicrograph shows epidermis (E), hair follicles (F), dermis (D) and hypodermis (H), containing small, patent signet-ring capillaries (arrows); main longitudinal artery (A) and vein (V); (**b**) Section taken from the centre of the treatment area of a mouse tail 24 h after PDT with 180 J cm$^{-2}$ of light. Note the damaged epidermis (E), artery (A) and vein (V), and especially the severely congested capillaries (arrows); (**c**) Section taken from the centre of the treatment area 5 days after PDT with 90 J cm$^{-2}$ of light. Note residual oedema (O) and patency of the numerous small blood vessels (arrows).

*Number of blood vessels with a cross-sectional area of 100–1,000 μm²* (Figure 4) In the dermis, following both PDT doses there was a significant increase in the number of vessels falling into this range by day 1 compared with the control group. There was a further significant rise between days 1 and 5 at both dose levels. At day 5 differences between the 90 and 180 J cm$^{-2}$ groups were significant.

The pattern in the hypodermis was similar to that seen in the dermis, with significant increases in the number of vessels by day 1 in both the dose groups compared with the controls and further significant rises at both doses by day 5.

*Number of blood vessels with a cross-sectional area of <100 μm²* (Figure 5) Differences between the two dose groups were particularly marked in these small vessels in the dermis. There was a significant increase in the number of vessels between days 1 and 5 in the treatment group which received 90 J cm$^{-2}$ but no corresponding increase in the group which received 180 J cm$^{-2}$. Thus, while there was no statistical difference between treatment groups on day 1, by day 5 the 90 J cm$^{-2}$ group had significantly more vessels.

The number of small blood vessels in the hypodermis on day 5 following PDT with 90 J cm$^{-2}$ was greater than the

**Table I** Absolute area occupied by different tissues in a cross-section of tails from untreated control mice or from mice treated 1 or 5 days previously by PDT

| | | *Area occupied by tissue* ($mm^2$) | | | | |
|---|---|---|---|---|---|---|
| | | | | *Time after PDT* | | |
| *Tissue* | *Dose* ($J\,cm^{-2}$) | *Untreated controls* | | *1 day* | | *5 days* |
| Epidermis | 90 | 0.24±0.02 | S | 0.42±0.02 | | 0.43±0.04 |
| | 180 | | | 0.41±0.02 | | 0.46±0.02 |
| Dermis | 90 | 1.20±0.06 | S | 1.36±0.09 | | 1.52±0.08 |
| | 180 | | | 1.44±0.09 | | 1.53±0.04 |
| Hypodermis | 90 | 0.96±0.06 | S | 1.31±0.08 | | 1.59±0.17 |
| | 180 | | | 1.24±0.12 | | 1.39±0.12 |
| Tendon | 90 | 1.74±0.17 | NS | 1.66±0.23 | | 1.78±0.22 |
| | 180 | | | 1.57±0.23 | | 1.74±0.17 |
| Bone | 90 | 0.83±0.14 | NS | 0.98±0.13 | | 0.75±0.12 |
| | 180 | | | 1.13±0.13 | | 0.76±0.13 |
| *Blood vessels* | | | | | | |
| Dermis | 90 | 0.0163±0.0022 | S | 0.0367±0.0049 | s | 0.0714±0.0106 |
| | 180 | | | 0.0317±0.0031 | s | 0.0550±0.0056 |
| Hypodermis | 90 | 0.0403±0.0071 | 1 day, NS | 0.0486±0.0050 | s | 0.0933±0.0096 |
| | 180 | | 5 days, S | 0.0492±0.0054 | s | 0.1055±0.0103 |

S, Significant difference between controls and treatment groups; NS, insignificant difference. Within treatment groups, s indicates a significant difference between horizontal or vertical pairs of data; where not so indicated, differences are insignificant.

number in the control and all other treatment groups. Therefore, in animals treated with 90 J cm$^{-2}$ there was a significant increase between days 1 and 5, while there was *no* significant change in animals treated with 180 J cm$^{-2}$.

### Discussion

We have shown previously that the probability of a tail healing or necrosing is related to the capacity for recovery of blood flow, measured functionally, after the combination of TPPS plus light (drug alone or light alone having no effect; Benstead & Moore, 1988*a*). These earlier experiments indicated that the probability of tail necrosis occurring in B6D2F$_1$ mice after 2 mg TPPS plus light would be low after 90 J cm$^{-2}$, and around 100% after 180 J cm$^{-2}$. At the lower, 'tolerance' dose, although one expects a reduction in blood flow on day 1, flow should return to normal by day 5. At the higher dose, however, no such improvement in flow between days 1 and 5 occurs. Where recovery in flow *does* occur, several mechanisms may contribute: decrease in tissue levels of vasoactive substances released by mast cells, recanalisation of existing vessels blocked by thrombus and the formation of new vessels. In the present paper we have attempted to relate previously observed functional effects to histological changes occurring in the tail over the same period, and in particular to quantitative aspects of the tail vasculature.

Regarding the methodology of these experiments, a decision was made to count *all* the blood vessels around a cross-section of the tail. The mice were prone in the jig and therefore the dorsal surface of the tail was closest to the light beam and might have been expected to receive the highest dose, and also to shield the ventral surface. In practice, reflection of light from the tube containing the tail and rotational movements of the tail during treatment meant that histological inspection failed to reveal 'hot' and 'cold' spots of damage in the 200 $\mu$m thick skin. Additionally, counting all vessels in the cross-section was felt to be more relevant to the necrosis end-point used previously, i.e. *complete* loss of tail resulting from full-thickness necrosis around the whole circumference (with no evidence of early focal necrosis in the dorsal region).

The increase in the cross-sectional area of the epidermis, dermis and hypodermis at days 1 and 5 following PDT at both light doses (Table I) is a measure of oedema formation. This has been noted in several studies previously, e.g. in normal cerebral tissue in mice (Berenbaum *et al.*, 1986), in the ears of mice (Lim *et al.*, 1986) and in mouse tails (Moore *et al.*, 1986). There was no significant difference in the increases between those animals which had received 90 J cm$^{-2}$ and those which had received 180 J cm$^{-2}$, despite the fact that one would expect a very low necrosis rate in the former group and 100% necrosis in the latter. Therefore vascular permeability as reflected in oedema formation does not follow the same light-dose–response curve as tail necrosis. This is in agreement with measurements of gross tail volume following graded doses of light (Moore *et al.*, 1986).

There was increase in the cross-sectional area of the vascular system following PDT, which was observed at both dose levels. This could simply be a reflection of the vasodilatation that occurs in pre-existing vessels. A count of the absolute number of blood vessels, however, revealed that by day 5 there was a significant increase in the animals treated with 90 J cm$^{-2}$ but *not* in those treated with 180 J cm$^{-2}$. This might imply that angiogenesis plays a role in the recovery of blood flow observed following 'low' doses of PDT and that it might be important in the prevention of necrosis.

To verify these conclusions, we further analysed the results according to the size of the blood vessels. Angiogenesis occurs by the division of viable endothelial cells at the capillary level (Ausprunk, 1979); therefore if the changes between days 1 and 5 were due to angiogenesis the increase should have occurred in the smaller blood vessels. The luminal diameter of true capillaries ranges from 3 to 10 $\mu$m, the upper limit being determined by the ultrastructure of the capillary wall, which consists of a single layer of endothelial cells, a basal lamina and an occasional pericyte (Rhodin, 1974). A class with an upper limit of area of 100 $\mu m^2$ would therefore be expected to include all the capillaries (as well as possibly some small arterioles and post-capillary venules). Vasodilatation would be expected to produce an increase in the number of vessels in the *larger* classes at both dose levels but an increase in the number of *small* vessels would only be expected on day 5 following the 'low' dose of 90 J cm$^{-2}$ and this indeed was confirmed. An increase in the number of

**Figure 2** Mean values $\pm 1$ s.e. for a group of six mice of the total number of blood vessels in the dermis and hypodermis of a tail cross-section, taken from the centre of the treatment field. Values are for untreated control animals (shown at the zero time point) and for mice treated 1 or 5 days after illumination with $90\,J\,cm^{-2}$ (——) or $180\,J\,cm^{-2}$ (– – –), given 24 h after 2 mg TPPS.

**Figure 3** Mean values for the total number of blood vessels of cross-sectional area $> 1{,}000\,\mu m^2$ in the dermis and hypodermis of controls or PDT-treated mice. All other details as for Figure 2.

vessels falling into the 'medium' and 'large' categories, which presumably reflects the vessel damage produced by PDT reflected in vasodilatation, was observed at both dose levels; here, there was no significant difference between the two doses. This is in accord with the evidence from our experiments on blood flow using xenon clearance (Benstead & Moore, 1988*a*) the subnecrotic light doses will produce vascular damage, but that at these doses vascular recovery is possible. It is also in agreement with the observations made by Star *et al.* (1986), who reported almost complete recovery of normal tissue circulation, brought about by revascularisation as well as recovery from vasoconstriction, 4 days following PDT when mammary carcinomas were transplanted into the subcutis of rats in transparent observation chambers.

New capillaries could grow into the treated area of mouse tails from the adjacent untreated normal tissue. They could also grow from 'surviving' capillaries within the treated area. Experiments varying the length of tail treated with light during PDT (Benstead & Moore, unpublished work) showed that as the field size was decreased, the light dose required to produce a 50% incidence of tail necrosis significantly

**Figure 4** Mean values for the total number of blood vessels of cross-sectional area 1,000–100 $\mu m^2$ in the dermis and hypodermis of controls or PDT-treated mice. All other details as for Figure 2.

**Figure 5** Mean values for the total number of blood vessels of cross-sectional area $<100\,\mu m^2$ in the dermis and hypodermis of controls or PDT-treated mice. All other details as for Figure 2.

increased. This was particularly marked at short lengths, e.g. 0.5 cm. The effect might be due to simple diffusion of oxygen and nutrients from the untreated area into the damaged part of the tail but new vessel formation from untreated tissue growing into the treatment field might also provide a basis for this effect. Conversely, the increasing probability of necrosis with increasing light dose when the area of the treatment field is kept *constant* (Benstead & Moore, 1988*a*) may provide evidence for viable capillaries in the treated area also playing a role in the new vessel formation.

This work was supported by the Cancer Research Campaign.

### References

AUSPRUNK, D.H. (1979). Chemical messengers of the inflammatory process. In *Handbook of Inflammation 1*, Houck, J. (ed) p. 317. Elsevier: Amsterdam.

BENSTEAD, K. & MOORE, J.V. (1988*a*). Vascular function and the probability of skin necrosis after photodynamic therapy: an experimental study. *Br. J. Cancer*, **57**, 451.

BENSTEAD, K. & MOORE, J.V. (1988*b*). The effect of fractionation of light treatment on necrosis and vascular function of normal skin following photodynamic therapy. *Br. J. Cancer*, **58**, 301.

BERENBAUM, M.C., HALL, G.W. & HOYES, A.D. (1986). Cerebral photosensitisation by haematoprophyrin derivative. Evidence for an endothelial site of action. *Br. J. Cancer*, **53**, 81.

BICHER, H.I., HETZEL, F.W., VAUPEL, P. & SANDHU, T.S. (1981). Microcirculation modifications by localised microwave hyperthermia and hematoporphyrin phototherapy. *Bibl. Anat.*, **20**, 628.

BUGELSKI, P.J., PORTER, C.W. & DOUGHERTY, T.J. (1981). Autoradiographic distribution of hematoporphyrin derivative in normal and tumor tissue of the mouse. *Cancer Res.*, **41**, 4606.

CASTELLANI, A., PACE, G.P. & CONCIOLI, M. (1963). Photodynamic effect of haematoporphyrin on blood microcirculation. *J. Pathol. Bact.*, **86**, 99.

LIM, H.W., HAGAN, H. & GIGLI, I. (1986). Phototoxicity induced by haematoporphyrin derivative in C5 deficient, mast cell deficient and leukopenic mice. *Photochem. Photobiol.*, **44**, 175.

MOORE, J.V., KEENE, J.P. & LAND, E.J. (1986). Dose-response relationships for photodynamic injury to murine skin. *Br. J. Radiol.*, **59**, 257.

RHODIN, J.A.G. (1974). *Cardiovascular System in Histology, a Text and an Atlas*. Oxford University Press: Oxford.

SELMAN, S.H., KREIMER-BIRNBAUM, M., GOLDBLATT, P.J., ANDERSON, T.S., KECK, R.W. & BRITTON, S.L. (1985). Jejunal blood flow after exposure to light in rats injected with hematoporphyrin derivative. *Cancer Res.*, **45**, 6425.

SIEGEL, S. (1956). *Non Parametric Statistics for the Behavioural Sciences*. McGraw-Hill: New York.

STAR, W.M., MARIJNISSEN, J.P.A., VAN DEN BERG-BLOK, A.E., VERSTEEG, J.A.C., FRANKEN, K.A.P. & REINHOLD, H.S. (1986). Destruction of rat mammary tumour and normal tissue microcirculation by hematoporphyrin derivative photoradiation observed *in vivo* in sandwich observation chambers. *Cancer Res.*, **46**, 2532.

ZHOU, C., YANG, W., DING, Z. & 4 others (1985). The biological effects of photodynamic therapy on normal skin in mice – II. An electron microscope study. *Adv. Exp. Med. Biol.*, **193**, 111.